# The Role of Larval Nutrition in Shaping Pheromone Composition in Fall Armyworm

**DOI:** 10.1007/s10886-025-01636-9

**Published:** 2025-08-05

**Authors:** Rajendra Regmi, Rabia Ali, Sanjana Akter, Fazila Yousuf, Bishwo Mainali, Soo Jean Park

**Affiliations:** 1 Applied Biosciences, Faculty of Science and Engineering, Macquarie University, Sydney, 2109 Australia; 2 Faculty of Agriculture, Agriculture and Forestry University, Chitwan, 44209 Nepal

**Keywords:** Pheromone release, Larval diet, GC-MS, Headspace volatile, Pheromone gland extract, *Spodoptera frugiperda*

## Abstract

The fall armyworm (FAW), *Spodoptera frugiperda* (JE Smith), is polyphagous that enables it to develop on different hosts, likely has a host-induced impact on its developmental biology as well as pheromone composition and production. While the effect of geographic variation on the components of FAW pheromone has been extensively studied, fewer studies have explored the influence of larval diet on the FAW pheromone composition. We conducted a laboratory study that examined the pheromone composition and production of FAW reared on horticultural plants, both known and potential hosts, in Australia. FAW larvae were raised on sweetcorn leaves/kernel, bean leaves/pod, okra leaves/fruits, strawberry leaves, capsicum fruits/leaves, and artificial diet. The headspace and pheromone gland compounds were collected from female FAW from each diet and analysed by gas chromatography-mass spectrometry (GC-MS). FAW female produced four pheromonal compounds (*Z*)-7-dodecenyl acetate (*Z*7C12Ac), (*Z*)-9-dodecenyl acetate (*Z*9C12Ac), (*Z*)-9-tetradecenyl acetate (*Z*9C14Ac), and (*Z*)-11-hexadecenyl acetate (*Z*11C16Ac). FAW reared on capsicum and okra exhibited higher release levels of these pheromonal compounds, whereas those fed on sweetcorn, beans, strawberry, and artificial diet released lower amounts. Notably, analysis of pheromones extracted from the pheromone gland showed that larval diets had no effect on the female FAW pheromone components, except for *Z*11C16Ac which vary significantly. While these findings improve our understanding of FAW pheromone production physiology mediated by larval diets, this knowledge could potentially be utilized to improve or optimise pheromone lure to enhance their effectiveness in early detection, monitoring and management of FAW populations across diverse cropping systems.

**Supplementary Information:**

The online version contains supplementary material available at 10.1007/s10886-025-01636-9.

## Introduction

Chemical communication is essential for the survival and reproduction of insect species (Wyatt 2014; Cardé and Willis 2008; Blomquist and Vogt 2003). Pheromones, species-specific chemical signals released by an organism, play a critical role in influencing processes such as survival, reproduction, and social organisation (Yew and Chung 2015) in many insect species. Given their essential roles, variations in pheromone composition can have significant ecological consequences such as disrupted mating communication or reproductive isolation. The variation in insect pheromone is primarily driven by genetic factors (Groot et al. 2014) and often occurs de novo (Seybold and Tittiger 2003; Tittiger and Blomquist 2017; Zacek et al. 2013). The intraspecific variation in pheromone due to physiological and environmental factors has been documented in several studies, affecting both the amount of pheromone produced and its composition (Duménil et al. 2014; Wang et al. 2015; Henneken et al. 2017). Among different factors, diet is a crucial factor, as it typically influences an individual’s overall condition and capacity to synthesise pheromones (Henneken et al. 2017). While diet significantly impacts insect growth and developmental parameters such as larval growth rate, pupal weights, and development durations (Ali et al. 1990; Jin et al. 2020; Whitford et al. 1992), the chemical and nutritional quality of diet is known to influence insect pheromone production (Boppre and Scheider 1985; Merli et al. 2018; Ming and Lewis 2010).

In polyphagous moth pests, sexual communication is driven by a pheromone released by the female, which is detected by conspecific males over long distances (Greenfield 1981). One such example is the fall armyworm (FAW), *Spodoptera frugiperda* (JE Smith) (Lepidoptera: Noctuidae). FAW is reported to attack more than 350 host plants belonging to 76 different families (Montezano et al. 2018), and relies on sex pheromone for sexual communication. The sex pheromone of FAW consists of multiple compounds, including (*Z*)−7-dodecenyl acetate, (*Z*)−9-tetradecenyl acetate, and (*Z*)−11-hexadecenyl acetate (Cruz-Esteban et al. 2018; Jiang et al. 2022; Tumlinson et al. 1986). Of note, the composition and proportion of these pheromone components vary among different geographic populations of FAW (Batista-Pereira et al. 2006; Groot et al. 2008; Jiang et al. 2022; Sekul and Sparks 1976; Tumlinson et al. 1986). Additionally, the two host strains of FAW, the corn strain mainly associated with corn, sorghum, and cotton; and the rice strain typically found on rice, turf, and Bermuda grass (Murua et al. 2015; Nagoshi et al. 2007; Pashley 1988), exhibit consistent differences in their female sex pheromone blends (Groot et al. 2008). The Australian FAW population is believed to be a hybrid of rice and corn strains (Piggott et al. 2021), and to date there is only one report on the pheromone compounds of the Western Australian population of FAW (Akter et al. 2025).

While numerous studies have explored geographical and strain-specific variation in the FAW pheromone production and composition, host-associated (host specific) variation remains unstudied in this species, including Australian populations. To address this research gap, we investigated the variations in sex pheromone composition and production of Australian population of FAW reared on different natural diets, including known and potential (= based on FAW’s prevalence reported by growers) hosts, during the larval stage under laboratory conditions. This information will be crucial for understanding host-mediated pheromone variation, assessing reproductive success, and laying the foundation for development of tailored pheromone blends that are more efficient to specific FAW populations in different cropping systems or regions.

## Methods and Materials

### FAW Colony

FAW eggs were sourced from the Commonwealth Scientific and Industrial Research Organisation (CSIRO) in Canberra, Australian Capital Territory, Australia, in March 2022; collected initially from maize crops in Western Australia. The insect colony was maintained under a controlled environment room (CER) of 25 ± 1 °C, 65 ± 5% relative humidity (RH), and with a photocycle of light: dusk: dark: dawn (13.5: 0.5: 9.5: 0.5 h). The larvae were reared on an artificial diet using the method of Apirajkamol et al. (2023). Adult moths were fed on a 10% (v/v) honey water solution (Capilano Pure honey 250 gm). All the experiments were conducted using the F10 generation.

### Host Plants

The hosts tested in this study were either major known hosts or potential hosts as reported by the growers (personal communication). Our preliminary observations indicate that older FAW instars prefer mature plant structures such as sweetcorn kernels, bean pods, and capsicum fruits over leaves. To support larval development and survival, we adjusted the diet by offering tender tissues (e.g., leaves) to early instars and mature tissues to older instars. This approach aligns with previous studies showing that early instars perform better on soft tissues, while later instars, with stronger mandibles, can feed effectively on tougher plant parts (Ali et al. 1990; Pannuti et al. 2015). For example, third instar larvae were provided with bean pods.

Seeds of sweetcorn (D.T. Brown -Kelvedon Glory F1), bean (Sabrini Foods Pty Ltd), and okra (D.T. Brown) were purchased from the supermarket and seeded in the flower pot (10.5 cm diameter ×14 cm height) using potting mixture (Scotts Osmocote 25 L Premium Potting Mix). The young leaves (3–4 weeks old) of sweetcorn, bean, and okra were harvested and used for the experiment. The petioles or mid-vein of the leaves were wrapped with moist cotton to prevent moisture loss. However, sweetcorn leaves were not wrapped because sweetcorn leaves did not dry quickly compared to other leaves (Acharya et al. 2022; Wu et al. 2021). Also, organic sweetcorn cob, bean pod, okra fruits, strawberry plants (12–16 weeks old), capsicum fruits, and capsicum plants (12–16 weeks old) were purchased from a local grocery store in Sydney, New South Wales, Australia.

### Larva Rearing

The neonate larva was seeded onto six different treatments, including five natural hosts (sweetcorn leaves/kernel, okra leaves/fruits, strawberry leaves, bean leaves/pods, capsicum leaves/fruits) and one artificial diet (using the method of Apirajkamol et al. (2023)) in an individual Petri plate^®^ (60 mm × 14 mm). The larvae were reared in the CER at 25 ± 1 °C with 65 ± 5% relative humidity with a photocycle of light: dusk: dark: dawn (13.5: 0.5: 9.5: 0.5 h).

The plant materials (leaves and fruits) were washed thoroughly with sterilised water, and excess water was removed using a sterilised paper towel. Each Petri plate with diet contained a single larva. For sweetcorn, fresh leaves were provided to the first two instars of the larvae, while 2–3 soft, young kernels were supplied to third instars larvae until pupation. In the case of beans, fresh leaves and a 1 cm long bean pod were given to the first two instars, whereas only bean pods measuring 1.5–2.5 cm were provided to the third instars larvae until pupation. Okra fresh leaves and 1 cm long okra fruits were consistently offered for all larval instars. For capsicum, leaves and 0.5 cm² pieces of fruit were provided during the first two instars, while 1.5–2.5 cm² pieces of fruit were supplied to the later instars. Strawberry leaves but no fruits were provided throughout all larval stages as their prevalence was mostly found on leaves, and seldom on fruits (Liburd and Rhodes 2018; personal communication with growers). The artificial diet block, measuring 2 cm × 2 cm × 1 cm, was placed in individual Petri plate^®^ for the artificial diet treatment. To maintain the quality of the diets, all food materials were replaced every 24 h, and any unused diets from previous days were removed. `A camel hair brush was used to move larvae while cleaning and replacing the food on each Petri plate. Daily inspections were conducted, and the artificial diet was replenished or supplemented before it finished or started to dry out. The larvae were allowed to pupate in the same Petri plate^®^. The feeding assay continued until the larvae pupated.

### Moth Rearing

Female and male pupae of each diet from each date were kept on a Petri plate^®^ (90 mm × 14 mm) inside the cage (12.5 L, 25 cm × 25 cm× 26 cm, Decor, Bunnings Warehouse, Australia) separately until emergence. Adult female moths that emerged on the same day were kept in the same cage (12.5 L, 25 cm × 25 cm × 26 cm, Decor, Bunnings Warehouse, Australia) (15 adult females in each cage). They were supplied with a 10% (v/v) honey water solution. A 3-day-old adult virgin female was used to extract the pheromone gland and headspace volatile collection in a room with the same photocycle and temperature as the rearing room. We observed everted pheromone glands of all females used in both headspace and gland extracts prior to each experiment.

### Headspace Volatiles Collection

A 3-day-old adult virgin female was kept in a gastight headspace vial (20 mL, 75.5 mm × 22.5 mm, with an 18 mm magnetic screw cap) at the onset of the dusk period, and the vial was sealed with a steel screw cap (Shimadzu Corporation, Nakagyo-ku, Kyoto, Japan). The vials were kept in the CER with the same photocycle, temperature, and humidity as the rearing room for 4.5 h to allow the release of pheromones. Pheromone collection was terminated by placing the vials at −30 °C freezer immediately after the 4.5 h period. The vials were stored in the same freezer for no more than three months before being analysed by GC-MS. For FAW reared on okra, we collected a total of 12 replications, whereas for all other treatments, we collected 13 replications.

### Analysis of Headspace Volatiles by Automated SPME-GC-MS

Pheromone release was assessed using solid-phase microextraction (SPME) followed by GC-MS analysis. An internal standard (1-octanol) was included in each vial to normalise GC responses and minimise variability associated with sampling and injection. When all other variables (e.g., age, sampling time, and analytical settings) are held constant, between-group comparisons based on internal standard normalisation remain scientifically valid. The use of a consistent internal standard also helped minimise technical variability associated with SPME fibre adsorption efficiency and GC-MS desorption or inlet variability. This semi-quantitative approach allowed for comparisons of pheromone release between dietary treatments. Although SPME is known to exhibit mass discrimination, 1-octanol provided highly consistent GC responses across all runs, validated by additional runs. This empirical stability, achieved through precise control of adsorption times and thermal conditions, allowed for reliable semi-quantification of pheromone components between treatment groups (Validation parameters are provided Table S1 in Supplementary materials). A stock solution of 1-octanol was prepared by dissolving 19.99 mg in reverse osmosis (RO) water in a volumetric flask (100 mL) to prepare a stock solution of internal standard (0.20 mg/mL), which was further diluted to give 0.04 mg/mL solution for sample analysis. An aqueous solution of 1-octanol was chosen due to the negligible affinity of SPME fibres for water, that has been utilised in various solid phase microextraction (SPME) analyses (Stiles et al. 2008). The solubility of 1-octanol, 0.49 mg/mL (Segatin and Klofutar 2004), adequately covers the analytical concentration range of the internal standard.

Analyses were performed on a Shimadzu GC-2030 gas chromatograph and Shimadzu GCMS-QP2020 NX mass spectrometer (Shimadzu Corporation, Nakagyo-ku, Kyoto, Japan) equipped with an AOC-6000 autosampler and a splitless/split injector. A Shimadzu SH-Rtx-5Sil MS fused silica capillary column (30 m × 0.25 mm, 0.25 μm) film was used. Helium (BOC, North Ryde, NSW, Australia) was used as the carrier gas, with a purity of 99.999% (Grade 5.0) and a constant flow rate of 1.49 mL/min.

SPME Arrow (DVB/PDMS, phase thickness: 120 μm, Shimadzu Corporation, Kyoto, Japan) was used for the adsorption of pheromone compounds and the internal standard. The autosampler parameters were maintained as follows: initially, the fiber was conditioned for 5 min at 260 °C; the sample vial was incubated for 5 min at 60 °C; the agitator speed was 250 rpm; the stirrer speed was 1000 rpm; the sample adsorption time was 5 min; the internal standard adsorption time was 1.8 s; and the sample desorption time was 1.5 min.

The samples were analysed in split mode with a 3:1 ratio. The initial column temperature was kept at 60 °C for 2 min, which was increased to 255 °C at a rate of 15 °C/min and held for 1 min. Both the injector and transfer line temperatures were set at 250 °C. The ionisation method was electron impact (EI) at 70 eV. The ion source temperature was 200 °C. MS was operated in Fast Automated Scan/SIM Type mode. SIM fragments were chosen based on the mass fragmentation patterns of each compound. The data was processed to obtain the peak areas of the target compound using GCMS Postrun Analysis software version 4.45. The peak areas of a target compound were normalised by dividing the peak area of a target by the peak areas of the internal standard. This normalisation enabled comparisons between groups. To validate the reliability of the internal standard in the method, we analysed an additional 11 and 11 female headspace samples for intraday and interday variations, respectively. These samples were collected using the same headspace volatile collection method described above (Table S1 in Supplementary materials).

### Pheromone Gland Extraction

The pheromone gland of a 3-day adult virgin female was excised between 4 h and 4.5 h of the scotophase. The pheromone gland was extracted from a live female moth by gently squeezing the tip of the abdomen and pulling out the pheromone gland with forceps under the stereoscope. The excised pheromone gland was collected in a 250 µL insert within a 2 mL glass vial kept on dry ice. Pheromone glands from each of the five females into a single insert served as one replicate. The vials were removed from dry ice, and 100 µL of hexane was added to the glands in the vial, which were kept for about 15 min at room temperature (25 °C). The pheromone gland extract was transferred to a new 250 µL glass insert within a 2 mL vial. The sample obtained was stored at −30 °C.

In preliminary trials, sets of one, three, and five glands were extracted as single replicates. While chemical signals were detected from the set of three glands, pooling five pheromone glands, used as a single replicate, per sample produced stronger GC signals that were more suitable for group comparisons. The gland extraction experiment utilised different numbers of replications based on the performance of the FAW on various hosts. Due to very low survival on capsicum, only one replicate was obtained, and it was excluded from the statistical analysis of gland extracts. For the other diet treatments, the number of replicates was as follows: five for beans and sweetcorn, four for the artificial diet, and three for okra and strawberry.

### Analysis of Pheromone Gland Extracts By GC-MS

(i) *GC-MS condition*. The general GC-MS conditions and EI as ionisation were the same as in the headspace analysis above, except for the temperature program. The initial column temperature was set at 80 °C, which was increased to 240 °C at a rate of 10 °C/min. The temperature was kept for 2 min and increased to 300 °C at 30 °C/min. GC-MS also analysed the natural gland extracts with chemical ionisation (CI) for the qualitative confirmation of the pheromone components. The samples were analysed in positive ion mode with methane gas (ultrapure, BOC, Australia) as the reagent gas. All GC-MS conditions were the same as those used with EI, except that spectra were obtained over a mass range of m/z 200–400.
(ii) *Preparation of Standard Solutions.* The four-pheromone compounds namely, (*Z*)−7-dodecenyl acetate (*Z*7C12Ac) (90% *Z*-isomeric purity), (*Z*)−9-dodecenyl acetate (*Z*9C12Ac) (90% *Z*-isomeric purity), (*Z*)−9-tetradecenyl acetate (*Z*9C14Ac) (95% *Z-*isomeric purity), and (*Z*)−11-hexadecenyl acetate (*Z*11C16Ac) (98% *Z*-isomeric purity) (BLD Pharmatech Ltd., China) stock solution were prepared by dissolving 17.5 mg, 18.9 mg, 22.0 mg, and 22.3 mg, respectively in *n*-hexane in 10 mL volumetric flasks. The stock solution was further diluted to prepare the following concentrations: *Z*7C12Ac (1.8 µg/mL), *Z*9C12Ac (1.9 µg/mL), *Z*9C14Ac (22.0 µg/mL), *Z*11C16Ac (4.4 µg/mL). These secondary stock solutions were diluted to prepare the seven-point standard solutions with the concentration range as per their approximate proportions found in natural glands extracts: *Z*7C12Ac (0.03–1.8 µg/mL), *Z*9C12Ac (0.03–1.9 µg/mL), *Z*9C14Ac (0.17–22.0 µg/mL), *Z*11C16Ac (0.07–4.4 µg/mL). An internal standard, *n*-heptadecane (5.06 µg/mL) stock solution was prepared. An aliquot of 5 µL of the internal standard was combined with 100 µL of the standard solutions and samples to yield 0.24 µg/mL. The internal standard was spiked into the extract after sample preparation, as the method produced analyte solutions in a clean solvent, suitable for GC analysis with minimal matrix interference. This approach has been used in previous studies for quantification under similar conditions (e.g., Niculau et al. 2018). The calibration curves showed strong linearity and reasonable LOD and LOQ, as presented in Table S2 in Supplementary Materials.
(iii) *Quantification of the Pheromone Compounds via Standard Curves*. The prepared standard solutions were analysed using GC-MS with the method described above, which generated standard curves of the pheromone compounds. The concentration of each compound was inferred from the standard curves. Subsequently, the amount of each compound was estimated by taking account of the original volume (105 µL) and the number of moths (n = 5) in a sample.

### Data Analysis

The data recorded from the experiment was tabulated using MS Excel and analysed using R software (R 4.2.0). The data transformation was done for non-normal data using guidelines from the bestNormalize package of R. The transformed data, which met normality assumptions, were analysed using one-way ANOVA followed by the Tukey test (*p* < 0.05) for all datasets. However, bar graphs were generated using the original data. Correlation analysis was conducted using group means of pheromone gland content and group means of pheromone release across diet treatments. A regression coefficient was calculated to assess the relationships between these variables.

## Results

### Sex Pheromone of FAW Females

**Fig. 1:**
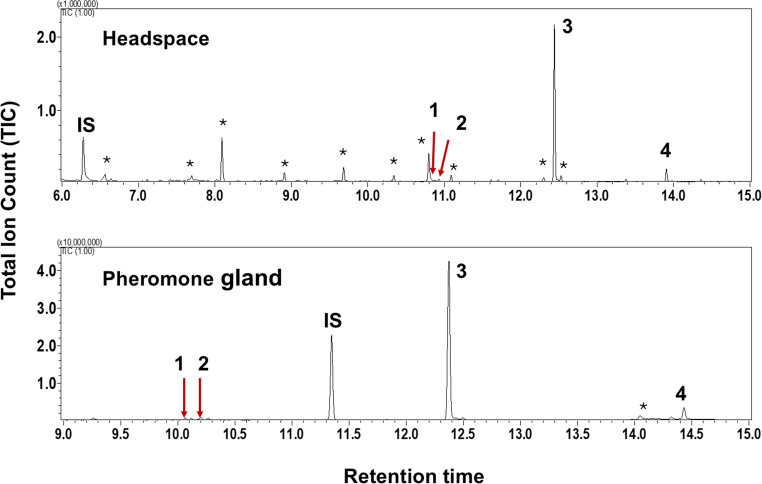
GC-MS chromatograms of volatiles collected from the headspace and pheromone gland extract of a calling FAW female. 1: (*Z*)−7-dodecenyl acetate (*Z*7C12Ac), 2: (*Z*)−9-dodecenyl acetate (*Z*9C12Ac), 3: (*Z*)−9-tetradecenyl acetate (*Z*9C14Ac), and 4: (*Z*)−11-hexadecenyl acetate (*Z*11C16Ac). IS: Internal standard (1-octanol in headspace; *n-*heptadecane in gland extracts). * represents impurities. The Y-axis represents Total Ion Count (TIC). The signal is scaled to a maximum of 1,000,000 for headspace samples and 10,000,000 for gland extracts, reflecting differences in compound abundance between sample types

The sex pheromone of females FAW was found to contain four compounds, including (*Z*)−7-dodecenyl acetate (*Z*7C12Ac), (*Z*)−9-dodecenyl acetate (*Z*9C12Ac), (*Z*)−9-tetradecenyl acetate (*Z*9C14Ac), and (*Z*)−11-hexadecenyl acetate (*Z*11C16Ac). The presence of these compounds was verified by comparing their retention times on a GC column and their mass fragmentation patterns with those of synthetic standards. *Z*9C14Ac was the predominant compound, while *Z*7C12Ac, *Z*9C12Ac, and *Z*11C16Ac were present in smaller amounts (Fig. 1). Additionally, chromatograms of headspace and gland extracts for each diet, including capsicum, which was removed from the statistic analysis of gland extracts, are provided in Fig S1 and S2, respectively, in Supplementary Materials. GC-MS analysis using chemical ionization (CI) confirmed each compound’s MH^+^ ion (protonated molecular ion). In contrast, molecular ions were not observed in the mass spectra obtained through EI ionisation. EI and CI mass spectra of the four pheromone components are provided in Fig S3 of the Supplementary Materials. Compounds marked with an asterisk in figures were also detected in blank runs under identical GC conditions and remained consistent across all samples, indicating they are background contaminants likely originating from the environment or GC system rather than being insect-derived.

### Headspace Volatile Collection

**Fig. 2:**
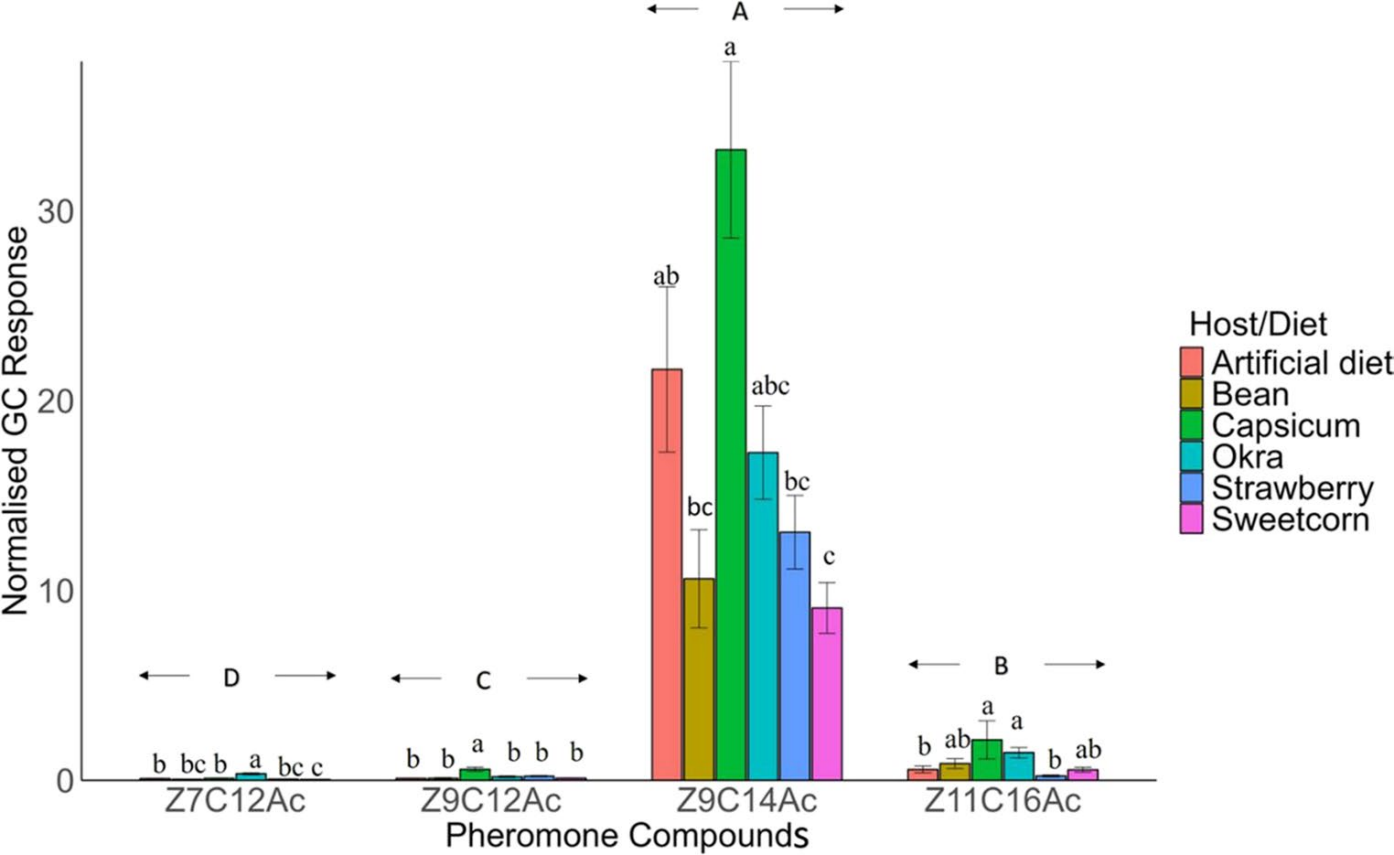
Normalised GC response of headspace volatile (Mean ± SE) released by FAW from the onset of the scotophase to 4.5 h into it. The Y-axis represents the GC peak area of each pheromone component normalised to the peak area of the internal standard (1-octanol), values are therefore unitless and represent relative emission levels. Data are semi-quantitative and intended for comparison between treatments, not absolute quantification. *ANOVA* was conducted, followed by *Tukey’s* post hoc analysis at *P* < 0.05 significance level. The different lowercase letters indicate significance at *P* < 0.05 for each compound, the different uppercase letters indicate significance at *P* < 0.05 among different compounds (*n* = 13 for each group of females except okra; *n* = 12 for okra). Error bars represent standard error calculated from the original (untransformed) data and are provided for visual interpretation only
(ii) *Relative Proportion of Normalised GC.* The relative proportion of four compounds released by female adult FAW from onset of the scotophase to 4.5 h into it on all the diets differed significantly (F_3,304_ = 214.50, *P* < 0.001) (Fig. 3). The highest proportion of *Z*9C14Ac (0.930) was released, followed by *Z*11C16Ac (0.048). The lowest proportion of *Z*7C12Ac (0.008) was released, followed by *Z*9C12Ac (0.014) (Fig. 3).

(i) *Normalised GC.* The levels of four compounds released by female adult FAW were significantly different (F_3,304_ = 192.5, *P* < 0.001), *Z*9C14Ac being the highest, followed by *Z*11C16Ac, whereas, *Z*7C12Ac and *Z*9C12Ac were the lowest (Fig. 2). Each compound released by FAW female adults reared on different host/diet during their larval stages were significantly different (*Z*7C12Ac, F_5,71_ = 18.22, *P* < 0.001; *Z*9C12Ac, F_5,71_ = 9.847, *P* < 0.001; *Z*9C14Ac, F_5,71_ = 8.611, *P* < 0.001; *Z*11C16Ac, F_5,71_ = 6.712, *P* < 0.001) (Fig. 2). The highest levels of *Z*9C12Ac were released by FAW reared on capsicum during the larval stage, compared to all other diets. For *Z*9C14Ac, the highest emission was also observed in FAW reared on capsicum, with similar levels found in those reared on artificial diet and okra. FAW reared on capsicum also released the highest amount of *Z*11C16Ac, similar to those reared on okra, bean, and sweetcorn.

**Fig. 3:**
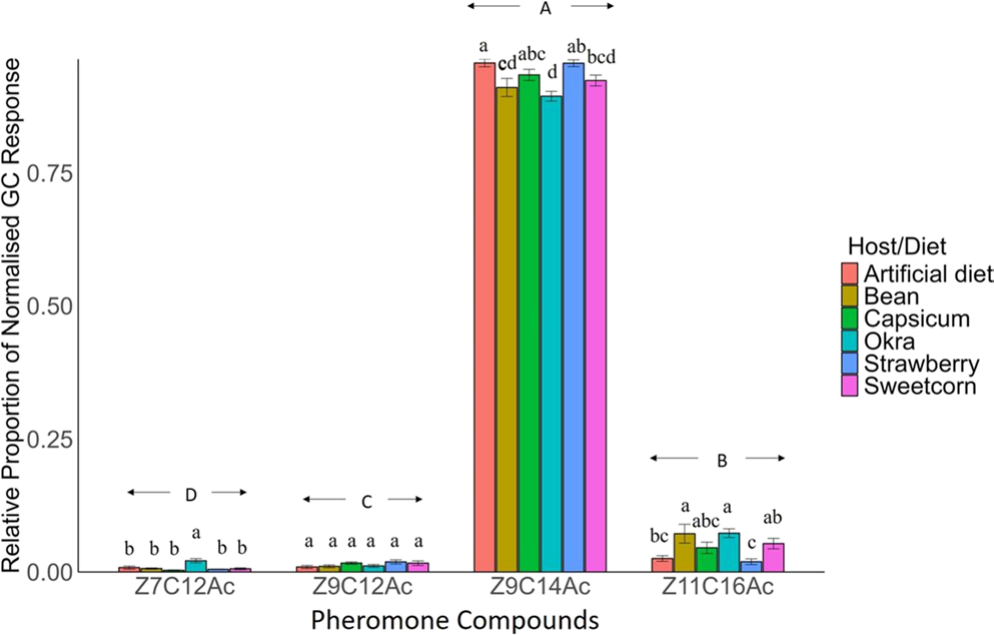
Relative proportion of volatile (Mean ± SE) released from the onset of the scotophase to 4.5 h into it. *ANOVA* was conducted, followed by *Tukey’s* post hoc analysis at *P* < 0.05 level of significance. The different lowercase letters indicate significance at *P* < 0.05 for each compound, the different uppercase letters indicate significance at *P* < 0.05 among different compounds (*n* = 13 for each group of females except okra; *n* = 12 for okra)

The relative proportion of three pheromonal compounds, *Z*7C12Ac, *Z*9C14Ac, and *Z*11C16Ac, were different (*Z*7C12Ac, F_5,71_ = 8.228, *P* < 0.001; *Z*9C14Ac, F_5,71_ = 7.318, *P* < 0.001; *Z*11C16Ac, F_5,71_=7.436, *P* < 0.001) among FAW reared on different host/diet at larval stage (Fig. 3). FAW released the highest relative amount of *Z*7C12Ac reared on okra compared to other hosts. FAW produced the highest relative amount of *Z*9C14Ac reared on an artificial diet, which was similar to capsicum and strawberry-reared FAW. The lowest relative amount of *Z*9C14Ac was produced by FAW reared on okra. FAW reared on okra produced the highest amount of *Z*11C16Ac, similar to FAW reared on bean and sweetcorn. The FAW reared on strawberry and artificial diet produced the lowest amount of *Z*11C16Ac. The relative amount of *Z*9C12Ac released by FAW reared on different hosts during the larval stage was statistically similar (F_5,71_ = 2.006, *P* = 0.088) (Fig. 3).

### Pheromone Gland Content

**Fig. 4:**
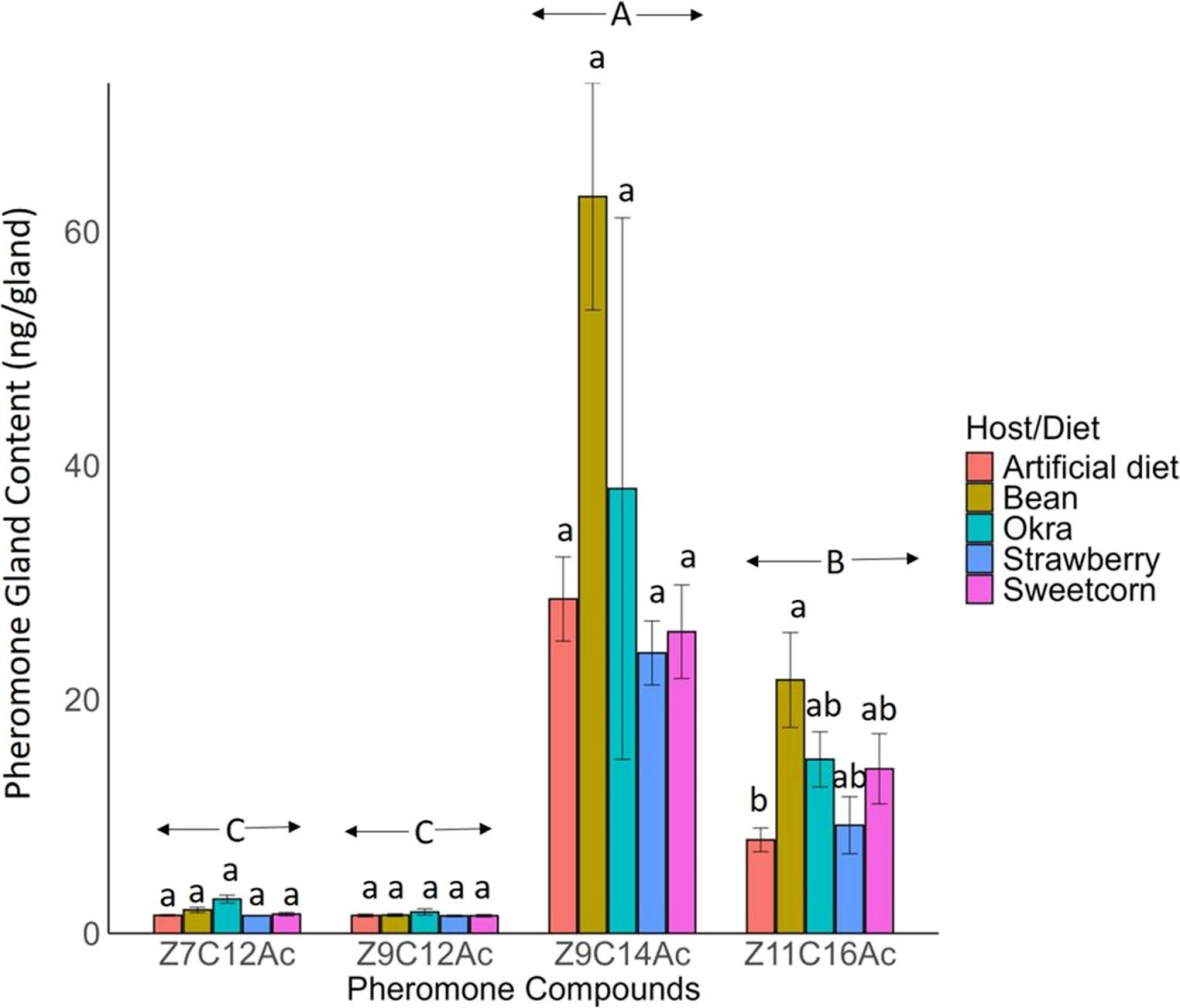
Amount of pheromone gland compounds (Mean ± SE) extracted from FAW female at 4 h to 4.5 h of scotophase. *ANOVA* was conducted, followed by *Tukey’s* post hoc analysis at *P* < 0.05 level of significance. The different lowercase letters indicate significance at *P* < 0.05 for each compound, the different uppercase letters indicate significance at *P* < 0.05 among different compounds (*n* = 4 for artificial diet; *n* = 5 for bean and sweetcorn; *n* = 3 for strawberry and okra)

The pheromone compounds produced in the pheromone glands of female FAW from all diets differed significantly (F_3__,76_ = 70.36, *P* < 0.001) (Fig. 4). *Z*9C14Ac was the most abundant (37.21 ng/gland), followed by *Z*11C16Ac (14.15 ng/gland), while *Z*7C12Ac (1.88 ng/gland) and *Z*9C12Ac (1.57 ng/gland) were the least abundant (Fig. 4). The amount of three pheromone compounds *Z*7C12Ac, *Z*9C12Ac, *Z*9C14Ac (*Z*7C12Ac, F_4,15_ = 2.94, *P* = 0.056; *Z*9C12Ac, F_4,15_ = 0.892, *P* = 0.492; *Z*9C14Ac, F_4,15_ = 1.539, *P* = 0.241) produced in gland of FAW reared on different host was similar, but not compound *Z*11C16Ac (*Z*11C16Ac F_4,15_ = 4.24, *P* = 0.017) (Fig. 4). FAW produced the highest amount of *Z*11C16Ac reared on bean (21.67 ng/gland) compared to those reared on an artificial diet (7.99 ng/gland). FAW reared on okra (14.88 ng/gland), sweetcorn (14.07 ng/gland), and strawberry (9.24 ng/gland) produced pheromones similar to those reared on both artificial diet and bean.

### Relative Proportion of Pheromone Gland Content

**Fig. 5:**
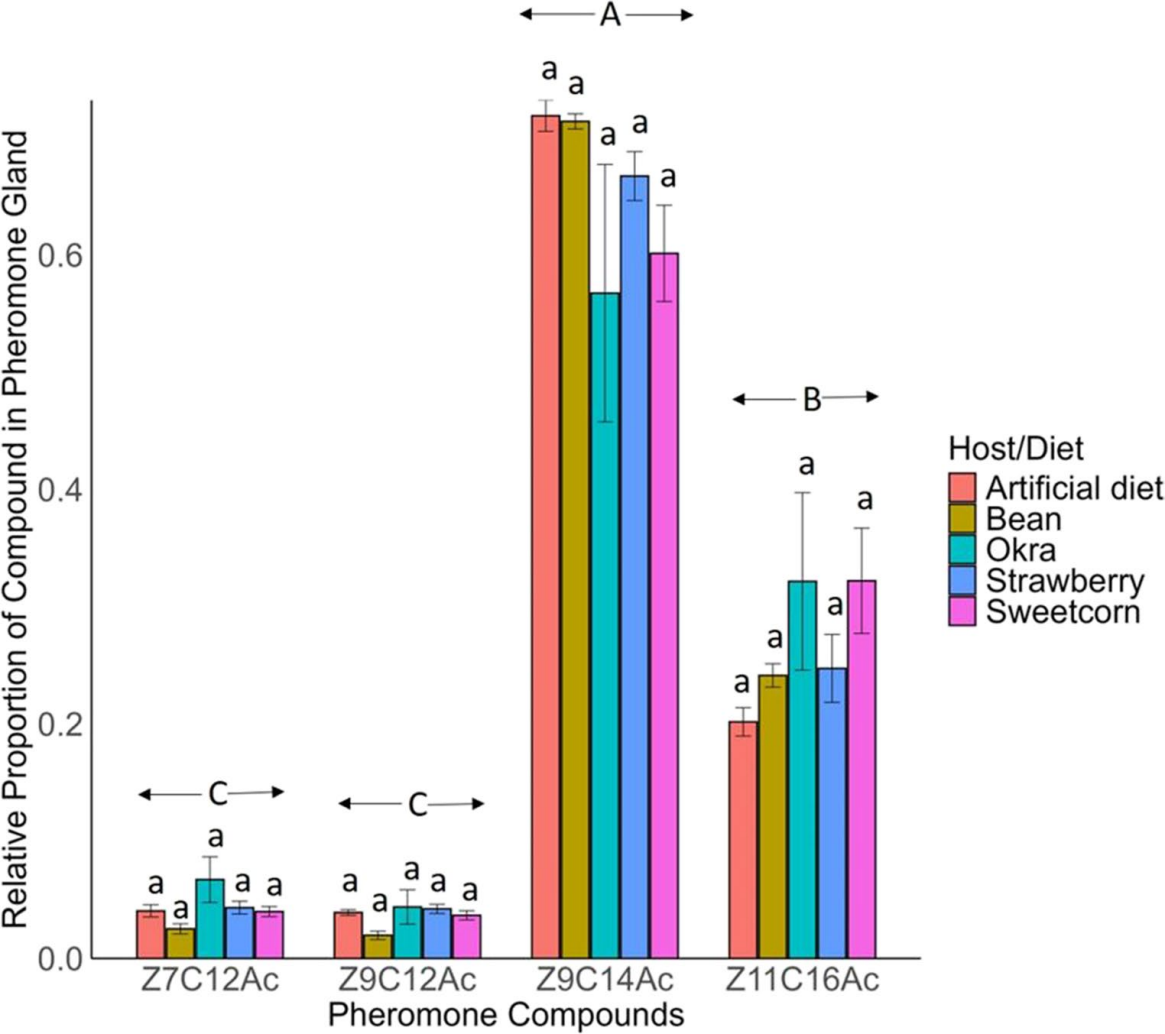
The relative proportion of pheromone gland compounds (Mean ± SE) extracted from FAW females at 4 h to 4.5 h of scotophase. *ANOVA* was conducted, followed by *Tukey’s* post hoc analysis at *P* < 0.05 level of significance. The same lowercase letters indicate non-significance at *P* ≥ 0.05 for each compound, the different uppercase letters indicate significance at *P* < 0.05 among different compounds (*n* = 4 for artificial diet; *n* = 5 for bean and sweetcorn; *n* = 3 for strawberry and okra)

The relative proportion of pheromone compounds produced in the pheromone gland of female adult FAW from all the diets collected was significantly different (F_3,76_ = 78.67, *P* < 0.001) (Fig. 5). The highest proportion of *Z*9C14Ac (0.658) was produced, followed by *Z*11C16Ac (0.266). The lowest proportion of *Z*7C12Ac (0.040) and *Z*9C12Ac (0.035) was produced in pheromone gland (Fig. 5).

The relative amount of all four-pheromone compound produced on gland of FAW reared on different host plants during its larval stage was similar (*Z*7C12Ac, F_4,15_ = 2.479, *P* = 0.089; *Z*9C12Ac, F_4,15_ = 3.060, *P* = 0.050; *Z*9C14Ac, F_4,15_ = 2.625, *P* = 0.077; *Z*11C16Ac, F_4,15_ = 2.023, *P* = 0.143) (Fig. 5).

### Relationship between Pheromone Gland Content and Release

**Fig. 6:**
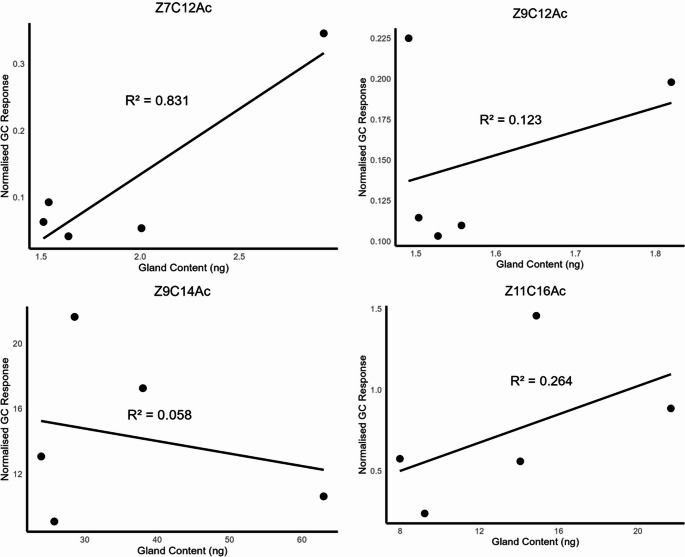
Linear regression between mean pheromone gland content and mean pheromone release across diet treatments. Each point represents a treatment-level mean based on separate groups of insects. Although individual insects differed between datasets, all experimental conditions were consistent. Results reflect treatment-level trends rather than individual-level correlations

A strong positive relationship was observed between the pheromone gland content and the release of *Z*7C12Ac, with R² = 0.831 (Fig. 6). In contrast, no significant correlation was found between gland content and pheromone release for the other three compounds tested. The relationship between gland content and release was weak for *Z*9C12Ac (R² = 0.123), Z9C14Ac (R² = 0.058), and Z11C16Ac (R² = 0.264) (Fig. 6).

## Discussion

We identified four sex pheromone components in the Western Australian FAW population: (*Z*)−9-tetradecenyl acetate (*Z*9C14Ac) as the dominant component, with (*Z*)−11-hexadecenyl acetate (*Z*11C16Ac), (*Z*)−7-dodecenyl acetate (*Z*7C12Ac), and (*Z*)−9-dodecenyl acetate (*Z*9C12Ac) as minor constituents. This pheromone profile is consistent with previous reports of FAW pheromone composition, where *Z*9C14Ac typically predominates and the other acetates serve as secondary components (e.g., Mitchell et al. 1985; Akter et al. 2025). Most global FAW populations share at least three of these pheromone components (*Z*7C12Ac, *Z*9C14Ac, *Z*11C16Ac) (Cruz-Esteban et al. 2018; Jiang et al. 2022; Tumlinson et al. 1986), though some regional populations include additional minor compounds (Batista-Pereira et al. 2006; Tabata et al. 2023). For example, a recent study in Japan identified a novel trace component, (*Z*,* E*)−9,12-tetradecadienyl acetate (*Z*9*E*12-14:Ac), in addition to the four compounds we observed (Tabata et al. 2023). Such geographic variation in pheromone profiles is well documented and likely reflects underlying genetic differences and local selection pressures in different populations.

We acknowledge that using SPME, while effective for compositional analysis, has certain limitations when used for quantification. Factors such as competition between analytes for fiber adsorption sites and differences in volatility and molecular weight can influence extraction efficiency and lead to biased representation of some compounds (Zhang et al. 2023; Diez-Simon et al. 2020). Additionally, SPME often operates under non-equilibrium conditions, which may limit the accuracy of absolute quantification (Zhang et al. 2023; Diez-Simon et al. 2020; Yang and Peppard 1994). However, in this study, our aim was not to determine absolute emission but rather to compare differences in pheromone release across diets. By standardising adsorption times, thermal conditions, and using an internal standard (1-octanol), we minimised technical variability and ensured reliable comparisons between groups. Given these considerations, SPME remains a suitable and widely accepted technique for semi-quantitative assessment of insect pheromones under controlled instrumental conditions (Sereshti et al. 2020).

In our Australian population, emitted pheromone was overwhelmingly *Z*9C14Ac (~ 93% of the total), with *Z*11C16Ac contributing ~ 5% and the two dodecenyl acetates together only ~ 2%. In the female pheromone gland, however, the composition was less skewed: *Z*9C14Ac comprised ~ 66%, *Z*11C16Ac ~ 27%, and *Z*7C12Ac and *Z*9C12Ac around 4% and 3.5%, respectively. Notably, the Western Australian FAW showed a higher proportion of *Z*11C16Ac relative to Z7C12Ac, a pattern similarly reported in a Mexican population (Cruz-Esteban et al. 2018). In contrast, Florida populations produce relatively more *Z*7C12Ac than *Z*11C16Ac (Tumlinson et al. 1986). This geographic variation in pheromone composition and ratios is influenced by genetic factors and local environmental conditions (Gao et al. 2020; Groot et al. 2009).

Similar to geographical variations, differences in larval diet influenced female FAW’s pheromone output in our study in terms of emission. Female FAWs that developed on capsicum and okra exhibited significantly higher emission of the major component *Z*9C14Ac and the minor component *Z*11C16Ac compared to females whose larvae fed on sweetcorn, bean, strawberry, or an artificial diet. Capsicum-reared females also released more of the minor component *Z*9C12Ac than those reared on the other hosts, suggesting the commonality of nutritional components of these hosts that may enhance pheromone production or release mechanisms. Notably, both capsicum and okra are rich in fatty acids with higher levels of palmitic, oleic, and linoleic acids (Silva et al. 2014; Souza Sora et al. 2015; Jarret et al. 2013; Sami et al. 2013; Berry 1980) compared to other diets in the study herein. Many lepidopteran sex pheromones with functional groups such as aldehydes, alcohols, and acetate esters are synthesised de novo from fatty acid precursors, (Choi et al. 2002; Jurenka 2024). Thus, lipid-rich larval diets (e.g., capsicum, okra) may increase the availability of these precursors, potentially enhancing adult pheromone biosynthesis and/or emission. However, because pheromone synthesis and release are regulated by complex and partially independent physiological processes (Foster 2022), diet may influence emission dynamics, such as calling frequency, duration, or metabolic energy availability, without necessarily altering the amount stored in the pheromone gland. This may explain why emission levels varied more strongly than glandular concentrations across dietary treatments. Previous studies have shown that larval diet quality can influence adult body size, energy reserves, and reproductive output of moths (Moreau et al. 2006; Colasurdo et al. 2009; Niitepõld and Boggs 2022), which in turn affect pheromone production and emission (Fujii et al. 2022; Ming and Lewis 2010). While our results indicate differences in pheromone emission, we did not measure calling frequency. Therefore, it remains unclear whether the observed variation is due to differences in calling frequency or a possible mismatch between the timing of pheromone collection and the peak emission across various treatments.

Previous studies across insect species show that larval diet quality can alter pheromone profiles– for example, *Utetheisa ornatrix* moths fed on seed-bearing plants produced more courtship pheromones; *Ceratitis capitata* fruit flies exhibited differences in pheromone quality and quantity based on larval substrates; and *Tribolium castaneum* beetles produced more aggregation pheromone when raised on a nutrient-rich diet (Conner et al. 1990; Merli et al. 2018; Ming and Lewis 2010). Of note, diet components are rarely converted directly into pheromone compounds; however, the overall nutritional quality of the diet can significantly impact a moth’s ability to produce pheromones (Henneken et al. 2017). Furthermore, dietary variation among individuals of the same species can lead to differences in pheromone production (Henneken et al. 2017).

The influence of larval diet on female moth sex pheromone production has been explored in various species, with mixed findings. In *Archips semiferanus* (oak leaf roller moth), Hendry et al. (1975) reported that certain chemicals in food plants may influence, in part, pheromones of the adult, even though the sex pheromone blend appeared consistent regardless of diet, as reported by Miller et al. (1976). Nevertheless, the broader hypothesis that plant-derived chemicals may influence adult pheromone profiles still holds potential and, as aforementioned, is supported in other taxa. Additionally, unlike earlier studies, which primarily focused on whether larvae sequester plant-derived compounds for adult chemical communication (e.g., Hendry et al. 1975; Miller et al. 1976), our study investigated how different larval diets influence the composition and relative abundance of sex pheromone compounds synthesized by adult females. Recently, Fujii et al. (2022) demonstrated a clear diet effect on pheromone titre of silkworm moths. They found that enriching the larval artificial diet with additional fatty acids resulted in significantly higher adult pheromone titers compared to a traditional mulberry leaf diet. This finding supports our observation that lipid-rich diets, such as capsicum and okra, can enhance pheromone production, reinforcing the idea that the availability of pheromone precursors in the diet can modulate pheromone biosynthesis in females. Although all individuals in our study were reared under consistent laboratory conditions, with larval diet as the only variable, the observed variability in pheromone release and content may reflect both diet-induced effects and inherent variation within the FAW population. Nevertheless, the controlled rearing environment supports the interpretation that diet is the primary factor driving the observed differences in pheromone profiles.

Interestingly, despite variations in the amounts of pheromone compounds released, the pheromone content within the glands remained largely similar across all larval diets, with the exception of *Z*11C16Ac. This suggests that females reared on different larval diets accumulated comparable pheromone quantities and maintained a consistent internal blend, even though the released (headspace) volatiles varied substantially. This contrasts with findings in *Helicoverpa armigera* (cotton bollworm), where Gao et al. (2020) reported that larval diet significantly influenced pheromone gland content. However, they also noted that the relative proportions of pheromone compounds remained consistent across diets, a finding that aligns with our study. The discrepancy between gland content and released volatiles in our results may be explained by differences in calling time, as McNeil and Delisle (1989) observed in *Pseudaletia unipuncta* reared on different host plants. However, Gao et al. (2020) found no effect of larval diet on female calling time on *H. armigera*. Of note, although larval diet influenced the overall pheromone release, the relative ratios of pheromone components remained unchanged in their production (gland) across treatments. This suggests that a single pheromone blend may be reliably used for monitoring or control across different host plant environments. Further research is needed to clarify whether diet-induced differences in FAW pheromone emission arise from changes in calling behaviour, release dynamics, or other physiological or environmental factors.

We also examined the relationship between the pheromone titer in the gland and the amount of pheromone released to assess whether females with higher internal pheromone stores emit proportionally greater amounts. Interestingly, this correspondence differed by compound. The one minor components *Z*7C12Ac showed a strong positive correlation between gland content and released amount. In other words, for this minor component, individuals with higher pheromone levels in their glands tended to release higher amounts, suggesting a direct linkage between synthesis/storage and emission. In contrast, we found weak, non-significant correlation for the dominant component *Z*9C14Ac (R² = 0.058) and other two minor components *Z*9C12Ac (R² = 0.123) and *Z*11C16Ac (R² = 0.264) This means that even moths with similar internal stores of *Z*9C14Ac or *Z*9C12Ac or *Z*11C16Ac could release very different amounts of those compounds, reinforcing the idea that release is under independent regulation (e.g., behavioral or neurophysiological control) for the major pheromone components. A similar study on gypsy moths found a uniformly positive correlation between pheromone gland content and emission rate (Giebultowicz et al. 1992). It is worth noting that our analysis was based on group-level means. While these results point to compound-specific release dynamics in FAW, confirmation would require analysis at the individual level.

Females reared on fatty acid rich host plants (like capsicum and okra) emitted significantly greater pheromone amounts than those reared on less optimal diets, even though their internal pheromone pools were similar. These findings suggest that larval diet primarily modulates the pheromone release process (e.g. through behavioural or metabolic cues) rather than altering the fundamental pheromone blend that the FAW female can synthesise. Our work provides a foundation for understanding pheromone composition in an Australian FAW population and highlights how diet-mediated differences could impact mating signals in this invasive pest. Although we found statistically significant differences in pheromone release across diets, the biological relevance of these differences remains uncertain. It is unclear whether the magnitude of these changes is sufficient to influence male attraction, as small variations in pheromone. However, previous work has demonstrated that FAW males distinguish subtle quantitative differences in blend composition, although their responses may also be influenced by other factors such as host plant volatiles (Unbehend et al. 2013). To confirm this, additional behavioural assays, such as wind tunnel or field experiments, would be necessary. Nonetheless, our findings suggest that larval diet can modulate pheromone output, which may have ecological and reproductive implications.

## Supplementary Information

Below is the link to the electronic supplementary material.

Supplementary Material 1 (DOCX 301 KB)

## Data Availability

No datasets were generated or analysed during the current study.
