# Supplementary material for: The Role of Larval Nutrition in Shaping Pheromone Composition in Fall Armyworm
Source: J Chem Ecol. 2025 Aug 5;51(4):79. doi: 10.1007/s10886-025-01636-9 (PMC12325486; doi:10.1007/s10886-025-01636-9)
Supplement: Supplementary file 1 — Supplementary Material 1 (DOCX 301 KB) [file 10886_2025_1636_MOESM1_ESM.docx]

**Supplementary Materials for:**

**THE ROLE OF LARVAL NUTRITION IN SHAPING PHEROMONE COMPOSITION IN FALL ARMYWORM**

RAJENDRA REGMI^1, 2*^, RABIA ALI^1^, SANJANA AKTER^1^, FAZILA YOUSUF^1^, BISHWO MAINALI^1^, SOO JEAN PARK^1^

*^1^Applied Biosciences, Faculty of Science and Engineering, Macquarie University, Sydney, Australia, 2109*

*^2^Faculty of Agriculture, Agriculture and Forestry University, Chitwan, Nepal, 44209*

Corresponding author email: rajendra.regmi@students.mq.edu.au


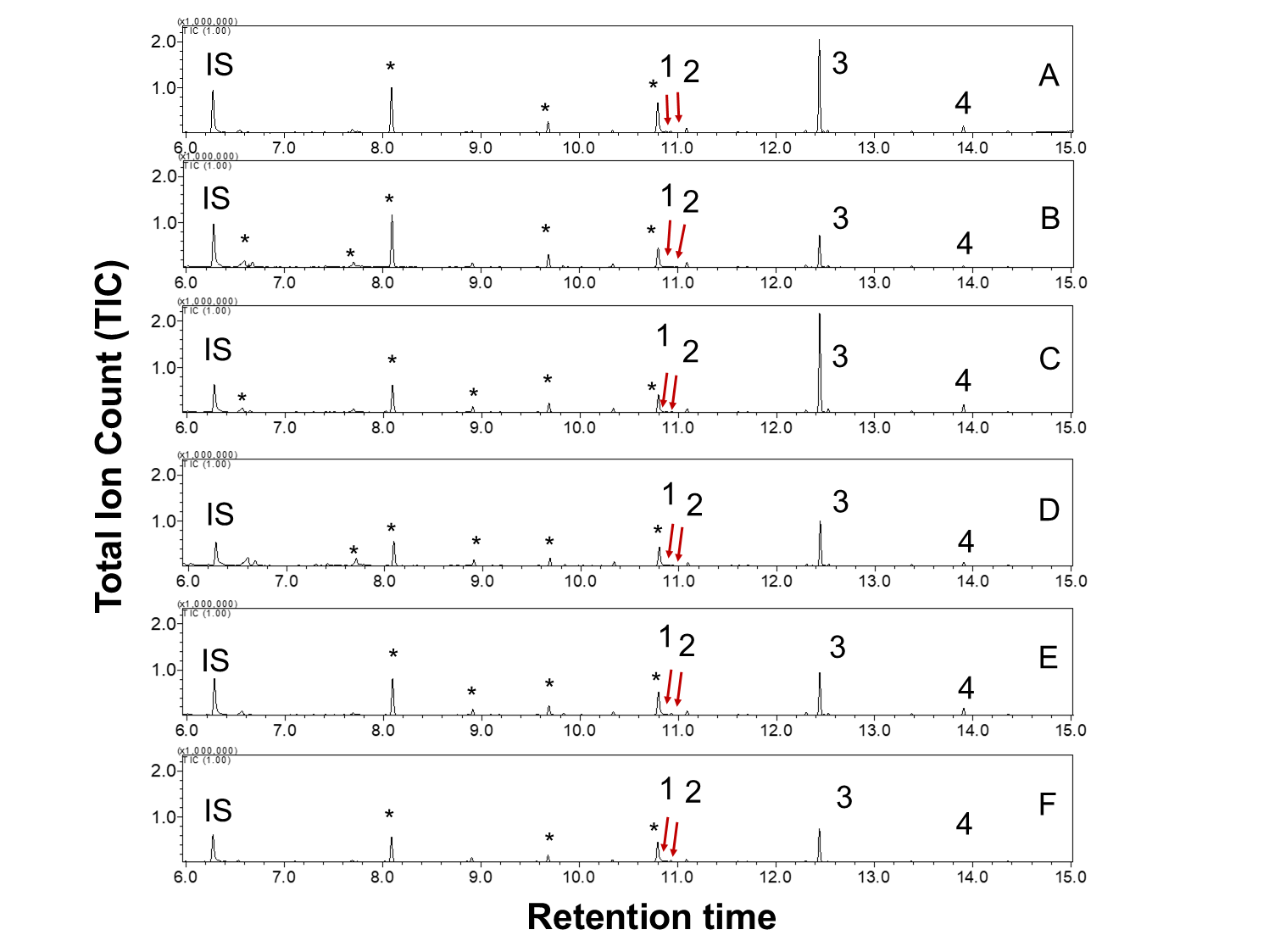


Fig. S1. Chromatograms of the headspace of calling females. A: artificial diet, B: bean, C: capsicum, D: okra, E: strawberry, F: sweetcorn, IS: internal standard (1-octanol). *: impurities.


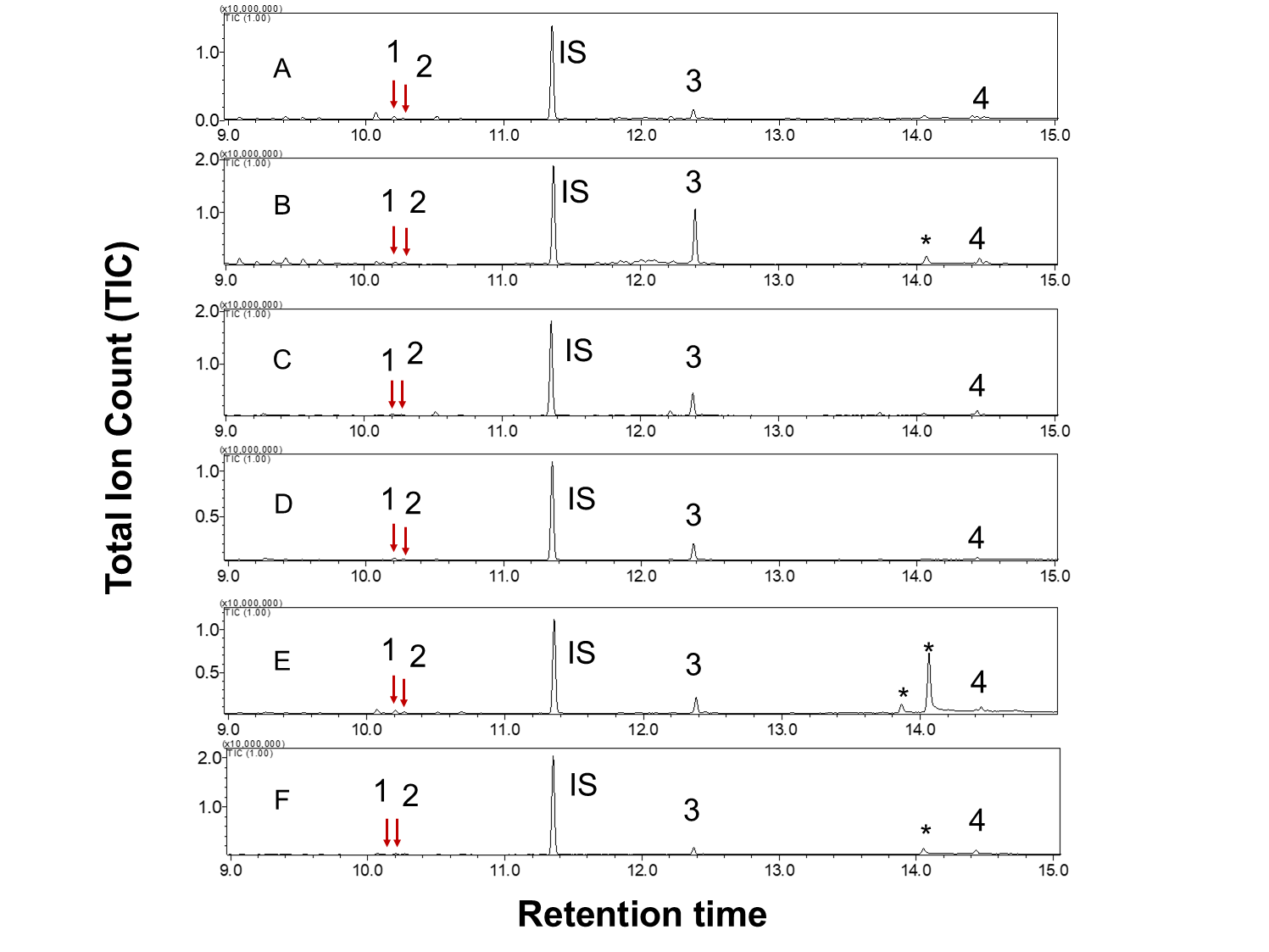


Fig. S2. Chromatograms of the pheromone gland extracts of calling females. A: artificial diet, B: bean, C: capsicum, D: okra, E: strawberry, F: sweetcorn. IS: internal standard (*n-*heptadecane). *: impurities.

1. Mass spectra of (*Z*)-7-dodecenyl acetate (molar mass: 226.3596)

EI

CI

MH^+^

1. Mass spectra of (*Z*)-9-dodecenyl acetate (molar mass: 226.3596)

EI

CI

MH^+^

1. Mass spectra of (*Z*)-9-tetradecenyl acetate (molar mass: 254.4081)

EI

MH^+^

1. Mass spectra of (*Z*)-11-hexadecenyl acetate (molar mass: 282.4614)

EI

MH^+^

Fig. S3. Mass spectra of (Z)-7-dodecenyl acetate, (Z)-9-dodecenyl acetate, (Z)-9-tetradecenyl acetate, and (Z)-11-hexadecenyl acetate analysed with electron impact (EI) and chemical ionisation (CI). MH^+^ represents protonated molecular ion

Table S1. Validation parameters for aqueous 1-octanol solution as the internal standard in headspace analysis. Additional samples were analyzed to confirm specificity and calculate relative standard deviation (RSD) values.

| Validation parameter | Observation/RSD |
| --- | --- |
| Specificity | No overlapping peaks observed |
| Reproducibility | 4.31% |
| Interday variation | 6.95% |
| Intraday variation | 8.67% |

Table S2. Linear regression equations, R^2^, linearity ranges, LOD and LOQ for (*Z*)-7-dodecenyl acetate, (*Z*)-9-dodecenyl acetate, (*Z*)-9-tetradecenyl acetate, and (Z)-11-hexadecenyl acetate used in gland analysis.

| Compound | Linear regression equation | R^2^ | Linearity range (µg/mL) | LOD  (µg/mL) | LOQ  (µg/mL) |
| --- | --- | --- | --- | --- | --- |
| (*Z*)-7-dodecenyl acetate | Y = (0.02194)X + (-0.00539) | 0.99776 | 0.02 – 1.80 | 0.01 | 0.02 |
| (*Z*)-9-dodecenyl acetate | Y = (0.03133)X + (-0.00737) | 0.99854 | 0.03 – 1.89 | 0.01 | 0.03 |
| (*Z*)-9-tetradecenyl acetate | Y = (0.03174)X + (-0.07335) | 0.99633 | 0.18 – 22.02 | 0.05 | 0.18 |
| (*Z*)-11-hexadecenyl acetate | Y = (0.01829)X + (-0.01326) | 0.99441 | 0.04 – 4.42 | 0.01 | 0.04 |
| The limit of detection (LOD) and limit of quantification (LOQ) for each pheromone component were determined based on the standard deviation of the response and the slope of the calibration curve. Specifically, LOD and LOQ were calculated using the following equations:  $LOD=\frac{3* \sigma}{S} and LOQ= \frac{10*\sigma}{S}$  where σ is the standard deviation of the response (y-intercepts) obtained from the calibration curve, and S is the slope of the corresponding curve. The values were derived from 3 replicates of low-concentration calibration standards. | | | | | |
